# Minimizing artifact-induced false-alarms for seizure detection in wearable EEG devices with gradient-boosted tree classifiers

**DOI:** 10.1038/s41598-024-52551-0

**Published:** 2024-02-05

**Authors:** Thorir Mar Ingolfsson, Simone Benatti, Xiaying Wang, Adriano Bernini, Pauline Ducouret, Philippe Ryvlin, Sandor Beniczky, Luca Benini, Andrea Cossettini

**Affiliations:** 1 ETH Zürich, D-ITET, 8092 Zürich, Switzerland; 2 University of Bologna, 40126 Bologna, Italy; 3 University of Modena and Reggio Emilia, 41121 Reggio Emilia, Italy; 4 University Hospital of Lausanne (CHUV), 1011 Lausanne, Switzerland; 5 Aarhus University Hospital, 8200 Aarhus, Denmark; 6 Danish Epilepsy Centre (Filadelfia), 4293 Dianalund, Denmark

**Keywords:** Biomedical engineering, Electrical and electronic engineering, Epilepsy

## Abstract

Electroencephalography (EEG) is widely used to monitor epileptic seizures, and standard clinical practice consists of monitoring patients in dedicated epilepsy monitoring units via video surveillance and cumbersome EEG caps. Such a setting is not compatible with long-term tracking under typical living conditions, thereby motivating the development of unobtrusive wearable solutions. However, wearable EEG devices present the challenges of fewer channels, restricted computational capabilities, and lower signal-to-noise ratio. Moreover, artifacts presenting morphological similarities to seizures act as major noise sources and can be misinterpreted as seizures. This paper presents a combined seizure and artifacts detection framework targeting wearable EEG devices based on Gradient Boosted Trees. The seizure detector achieves nearly zero false alarms with average sensitivity values of $65.27\%$ for 182 seizures from the CHB-MIT dataset and $57.26\%$ for 25 seizures from the private dataset with no preliminary artifact detection or removal. The artifact detector achieves a state-of-the-art accuracy of $93.95\%$ (on the TUH-EEG Artifact Corpus dataset). Integrating artifact and seizure detection significantly reduces false alarms—up to $96\%$ compared to standalone seizure detection. Optimized for a Parallel Ultra-Low Power platform, these algorithms enable extended monitoring with a battery lifespan reaching 300 h. These findings highlight the benefits of integrating artifact detection in wearable epilepsy monitoring devices to limit the number of false positives.

## Introduction

Epilepsy is a common neurological disorder that affects more than 50 million people worldwide^1^ and is characterized by the recurrence of seizures which temporarily compromise the function of the affected people’s brain. About one-third of persons with epilepsy (PWE) continue to suffer seizures despite receiving appropriate antiseizure medications. In most instances, these persons will lose awareness during their seizures, putting them at risk of accidents, traumatism, and even death. Furthermore, PWE will often fail to remember the occurrence of their seizures and will thus be unable to inform their physician to adjust therapy appropriately. These issues have led to an increasing interest in developing seizure detection solutions with two goals: (1) sending an alarm to family members or caregivers to protect PWE from the immediate risks entailed by seizures, (2) providing a reliable seizure count that they can share with their physicians to optimize treatment.

Several methods are being developed for this purpose; most currently rely on biosignals captured at the wrist or arm, including surface electromyography, 3D-accelerometry, electrodermal activity, and photoplethysmography. While several methods proved effective in detecting one subtype of seizures, i.e., generalized tonic-clonic seizures (GTCS), they fall short of detecting most other seizure types. For this reason, other methods attempt to detect seizures using the classic neurobiological hallmark of epileptic seizures, Electroencephalography (EEG). Indeed, by definition, a seizure reflects an abnormal EEG signal called an epileptic discharge. Diagnostic methods to perform short-term EEG recordings (i.e., from 20 min to several days) are very well established but not adapted to the purpose of chronic recordings over months or years to achieve the above-described objectives.

Indeed, conventional EEG systems are bulky and uncomfortable, causing patients to perceive stigmatization. Furthermore, the long wires used to connect multiple electrodes are a significant cause of motion artifacts on the EEG traces^2^. Consequently, wearable solutions are paramount for long-term continuous EEG monitoring^3^. In this context, the need for wearables-based long-term seizure detection requires empowering such solutions with seizure-detecting capabilities via Machine Learning (ML), to enable prompt interventions from caregivers during or immediately after the seizures, reducing their impact and providing more reliable information to the physicians (to optimize anti-seizure therapies)^4^. However, the development of EEG-based seizure detectors for wearable Internet of Things (IoT) devices is faced by multiple challenges, which we address in this paper.

First, most of the existing Artificial Intelligence (AI) models rely on a large number of electrodes^5^ (ideally all electrodes of standard EEG-caps): unobtrusive wearable solutions are limited to a lower number of channels, and they face the challenge of maintaining the same levels of performance as for full-channel systems. In addition, the impact of false alarms is much greater in long-term monitoring settings (since it relates to the willingness of patients to use the devices), ultimately resulting in the need to maximize specificity (also at the price of a lower sensitivity) as the main performance metric^6^.

Second, artifacts play a critical role in wearable IoT devices. While data acquired in epilepsy monitoring units (EMUs) usually are inspected by experts and feature a low amount of artifacts (which can be labeled)^7^, wearable EEG systems produce signals with low signal-to-noise ratio^8^ that are more affected by artifacts than full EEG-caps or implanted solutions. If not accounted for, artifacts can significantly increase the number of false alarms^9^, possibly making a wearable seizure detector not usable in practical settings (low specificity). Therefore, automated seizure detection frameworks must be combined with artifact detection (and, possibly, filtering).

Last but not least, wearable IoT devices must also fulfill the following requirements: (a) small and comfortable form factor; (b) long-battery life; (c) low latency. To ensure this, smart edge computing based on low-power microcontrollers (MCUs) has recently been introduced, proving its effectiveness in providing long-term operation and executing AI models^10^. However, the challenge is that the AI algorithms need to fit the computational capabilities of wearable devices. Consequently, the choice of models has to be narrowed down, so that they can be implemented on a low-power MCUs.

Seizure detection has received much interest in the past decades, and recently the usage of Deep Learning (DL) methods has increased considerably. The main metrics of interest are sensitivity (i.e., the ability to detect seizures) and false alarm rates (false positives, FP). The latter is essential since it relates to the trust and willingness of patients to use the devices (a maximum of one false alarm per day is typically considered acceptable)^6^. It is also worth noticing that not all papers report false alarm rates and report only specificity. Unfortunately a high specificity does not automatically translate to an excellent false alarm rate: for example, a specificity of 99% for 2s prediction windows results in 18 FP/h. In the following, we review the performance of published works that are compatible with deployment on wearable IoT devices, focusing on the false alarms and sensitivity metrics. False alarm rates (FP/h) are calculated as $\frac{3600}{W} \times (1-Sp)$, where *W* is the observation window (in seconds) and *Sp* is the reported specificity.

**Figure 1:**
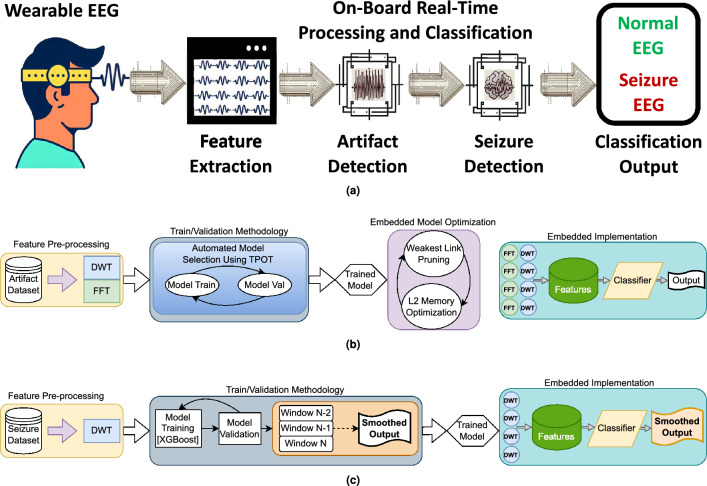
Proposed EEG artifact and seizure detection framework overview. (**a**) Provides a high-level view of the framework explored, encompassing the progression from raw EEG input, through artifact detection(rejection and smoothing of artifacts are also explored at this stage), to seizure detection, ending with the final output. (**b**) Details the artifact detection workflow: four temporal channels’ input data are preprocessed using Discrete Wavelet Transform (DWT) and Fast Fourier Transform (FFT); model selection is optimized with Tree-based Pipeline Optimization Tool (TPOT), and the selected model is pruned via the Minimal Cost-Complexity Pruning (MCCP) algorithm to fit the target processor. (**c**) Illustrates the Seizure Detection Workflow: DWT is utilized for preprocessing input data from the same four temporal channels; XGBoost performs classification into normal EEG and seizures; the classifier’s output is post-processed and smoothed using a majority vote (the smoothing is based on the last two predictions of the model, to preserve causality). Finally, the framework is executed on a parallel ultra-low power (PULP) platform, enabling energy-efficient, real-time processing and classification.

Sayeed et al.^11^ and Olokodana et al.^12^ present representative examples of seizure detection frameworks based on statistical feature extraction. However, both papers rely on an intracranial setup that requires invasive brain surgery and is therefore not applicable to wearable surface EEG systems. Samiee et al.^13^ proposed a novel feature extraction technique called rational discrete short-time Fourier Transform. However, their work also bases the experiments on intracranial data, and despite achieving good sensitivity ($97\%$), they have a false positive alarm rate of around 65 FP/h. In the study by Wang et al.^14^, the authors use a wavelet function decomposition and a directed transfer function to extract features, which are fed to a support vector machine classifier. Even though they achieve an excellent specificity of $99.5\%$, when considering their prediction window of 2s, the false alarm rate results in 9 FP/h. Fan et al.^15^ achieved very high sensitivity ($98.48\%$) and low latency results by means of temporal synchronization patterns (quantified using spectral graph theoretic features). However, their best-resulting model still features a very high false alarm rate of $21.4\%$ (over 270 FP/h considering their 1s prediction window).

Improvements of the false alarm rate are instead provided by a combination of spectral, spatial and temporal features and an SVM classifier of^7^, where a sensitivity of 96% and a false alarm rate of 0.08 FP/h was achieved. However, the proposed approach is not robust to the reduction of the number of channels^16^, thereby becoming unfeasible for a wearable edge implementation.

Gomez et al.^17^ utilize a fully connected convolutional neural architecture combined with a “First Seizure Model” (to achieve better performance on hard-performing subjects). Despite the low sensitivity (around 60%), they achieve a false alarm rate of 3.6 FP/h (relatively low compared to other works but still too high for practical usage). An alternative inspiring approach is reported by Baghersalimi et al.^18^, who propose a many-to-one signals knowledge distillation aiming at low-power applications. Their best-performing multi-modal model has a specificity of 95.66% (however resulting in 52 FP/h, when looking at their prediction interval of 3s).

In summary, while there is rich literature about EEG-based seizure detection, few works concentrate on low-channel-count scenarios and even fewer demonstrate low electrode count coupled with low false alarm rate^19,20^. *Hence, there is a strong need for seizure detectors based on a small number of channels capable of providing nearly-zero false alarms, with high sensitivity, and computational requirements that would fit the limited resources available on a wearable device.*

As stated before, a major problem in seizure detection is represented by EEG artifacts, some of which are often mistaken for seizures due to their morphological similarity in amplitude and frequency^21^. Main artifact sources are specifically ocular, cardiac, and (most frequently) muscular artifacts^22^. Many artifact detection/rejection algorithms have been proposed, relying on both supervised and unsupervised methods. The work of Khatwani et al.^23^ is a relevant example of artifact detection on an in-house dataset, where an energy-efficient convolutional neural network (CNN) processes raw EEG signals without the need for feature extraction and achieves around 74% accuracy.

Beyond private datasets, the Temple University Artifact Corpus (TUAR)^21^ is the most used reference for EEG artifacts analyses. Usage examples include the DL frameworks of Qendro et al.^24^, achieving a 0.838 F1 score, the DL model of Kim et al.^25^, which classifies artifacts with a 75% accuracy, and the convolutional-transformer hybrid network of Peh et al.^26^, achieving 0.876 Area Under the Receiver Operator Characteristic (AUC) score (by using a belief matching loss function at the cost of high computational costs).

Artifacts in EEG signals can also be smoothed with Blind Source Separation (BSS)^27^, which is the task of separating signal sources from a set of mixed signals. This is an inherently difficult problem and requires multiple assumptions and a recent study^28^ even suggests this should not be done. An example of a BSS technique is the Independent Component Analysis (ICA), which is a well-established approach for separating brain contributions and non-brain contributions from EEG signals.

ICA is a linear decomposition technique^29^ that relies on the fact that EEG signals are stationary, statistically independent, and originated from a non-Gaussian distribution. In ICA, Independent Components (ICs) are extracted from EEG signals and inspected (manually or automatically) to eliminate the noisy components. By reconstructing the EEG signal from the selected subset of ICs, the new EEG signal does not contain any artifacts. While ICA has been used successfully in the past to decompose multi-channel EEG recordings and clean artifacts from them, using ICA for wearable real-time usage faces serious challenges.

Firstly, ICA is an unsupervised algorithm that does not provide information on what the ICs represent in the original data. Consequently, manual inspection of all the ICs is needed, or a separate classifier has to be developed. Furthermore, ICA requires a large amount of data. As a rule of thumb^30^, the amount of data needed to get a reliable ICA decomposition is around $kn^2$ where *n* is the number of channels. Usually, $k>>20$ is required, or alternatively, a wider dataset is needed. However, online seizure detection methods typically rely on a few epochs, thereby ICA is inapplicable.

Finally, multi-channel EEG is required for ICA to decompose brain contributions and non-brain contributions. In fact, the signals in the sensors’ domain are required to be at least as large as the number of sources to be extracted. However, wearables feature a low channel count. As an example, decomposition on a 3-channel setup can at most extract 3 separate sources, and the separation of artifact (non-brain-components) and brain-component is not guaranteed to be clear enough. Approaches to exploit ICA with low channel count include the work of Rejer et al.^31^, where artificial signals are extracted from the original signals using a zero-phase filtering operation and are later separated in ICs. However, the continuous generation of artificial signals followed by a classifier is cumbersome for a wearable since it requires significant storage, power budget, and computation time.

While ICA is primarily used as an offline method, Artifact Subspace Reconstruction (ASR)^32^ is an artifact filtering technique that was designed for online operation. Similar to Principal Component Analysis (PCA), ASR works in the principal component space, compares short segments of EEG data to a statistical model (learned from clean calibration data), and then can filter out artifacts. ASR has been shown to be better than ICA at removing artifacts^33^ and Tsai et al.^34^ show that the use of an ASR technique improved the performance by up to $8.6\%$ on three distinct Brain–Computer Interface (BCI) tasks. The usage of ASR does require the calibration of user-defined parameters, also a multi-channel EEG system is usually recommended^35^. Still, recent studies such as by Cataldo et al.^36^ have shown that it can be effective even with a low channel count. Nevertheless, the continuous filtering of data is power-hungry for a wearable device, and as explored by Blum et al.^37^, a considerable amount of time and memory is needed. Therefore, also in the context of ASR, an artifact detector appears as necessary (to determine when filtering is necessary).

First attempts in combining seizure and artifact detection are reported by Islam et al.^38^, where artifacts and seizures are separated by means of a stationary wavelet transform, and in a later paper^39^, where mobility artifacts are removed from EEG data via ICA. However, both papers operate on offline data, with no online implementation. Additionally, the authors faced the challenge of the scarcity of publicly accessible datasets that are annotated with labeled seizures and artifacts, and thereby resorted to the combination of multiple data sources or the complete synthesis of data to overcome this issue. In this work we operate in a similar way and combine multiple EEG datasets for developing a combined artifacts and seizure detection.

Within this context, we present two frameworks, based upon boosted trees^40^, to accurately detect seizures and EEG artifacts. Furthermore, we demonstrate the importance of combining these two frameworks in a single seizure and artifact detection signal chain, and we implement it on a PULP-based edge device (GAP9) suitable for wearable unobtrusive epilepsy detection with a reduced electrode montage. Figure 1a summarizes the workflow explored and implemented in this paper. We release open source code under https://github.com/pulp-bio/Artifact-Seizure. Our contributions are the following:

- We extend upon a previous seizure detection framework^41^, further validating and improving the performance of the models by relying on Gradient Boosted Trees (XGBoost), also accounting for a larger number of patients (on the CHB-MIT dataset) and including analyses on a novel private dataset. Results demonstrate an average sensitivity of $65.27\%$ and $57.26\%$ for the two datasets, respectively, with a majority of patients (approximately $60\%$) experiencing no false positives.
- We extend upon a previous artifact detection framework^42^, improving its performance by relying on pruned Gradient Boosted Trees and achieving a state-of-the-art (SoA) accuracy score of $93.95\%$ and 0.838 F1 score on an artifacts dataset.
- We propose a method for combining these two frameworks in the absence of datasets labeled for both seizures and artifacts. By incorporating an artifact detection model prior to the seizure detection, we observed a substantial reduction in false positives per hour (FP/h), from 22.9 FP/h to 1.0 FP/h. Hence, the artifact detector filters out $96\%$ of the false alarms (or, conversely, the absence of the artifact detector results in $22.5\times$ more false alarms).
- We implement the proposed framework on the PULP-based GAP9 processor, demonstrating an average power envelope of 26.1 mW and energy consumption as low as 7.93 $\upmu$J per window feature extraction and classification, which leads to a battery lifetime of 300 h, very well suited for long-term monitoring in normal life conditions.

## Methods

### Seizure detection methodology

In our previous work^41^, we conducted an analysis on a subset of nine patients from the popular CHB-MIT dataset from the Children’s Hospital of Boston and MIT^43^. The dataset comprises data acquired at 256 samples per second at a 16 bit resolution, following the international 10–20 system of EEG electrode position, on 23 patients aged between 1.5 and 22 years old with intractable seizures. Our previous results on a limited number of patients demonstrated that utilizing a reduced electrode montage configuration did not degrade the efficacy of the proposed system, but improved outcomes for many patients. The findings of^41^ also highlighted the significance of considering subject-specific models in addition to global models, and the AdaBoost model (a variant of the boosted tree algorithm), emerged as the most effective method in this examination.

Starting from these results, here we expand our analysis and use all available EEG seizure recordings from the CHB-MIT dataset. Furthermore, we expand our investigations by exploring the performance of Gradient Boosted Trees, specifically XGBoost^44^, across all patients in the dataset while solely utilizing a reduced electrode montage or, more specifically, only the temporal electrodes (F7–T7, T7–P7, F8–T8, T8–P8) which is a convenient location to probe EEG signals with non-stigmatizing wearables^45^.

Finally, we also take into account that the CHB-MIT dataset presents some deviations from typical monitoring use cases (pediatric patients, gaps in the EEG traces, nearly zero artifacts). Thus, to further validate our approach, we also consider a novel private dataset curated by the Lausanne University Hospital (CHUV), which can be considered more representative of real-time monitoring scenarios in a practical clinical setup (adults, continuous monitoring, no gaps in the EEG data, broader inclusion criteria with patients at risk of presenting generalized tonic-clonic seizures recorded via video-EEG). The dataset derives from a project funded by the Swiss National Science Foundation (PEDESITE) and targets the development of novel wearable solutions for seizure detection. In the following, this dataset will be referred to as the PEDESITE dataset.

Figure 1c shows a sketch of the overall workflow of our investigations. In the following subsections we provide the details about the novel dataset and the methodology followed for the feature processing and classification tasks.

#### Dataset

We consider the well-known CHB-MIT dataset and the novel PEDESITE dataset for these analyses. The Pedesite study takes place during routine clinical evaluations at the in-hospital epilepsy monitoring unit where patients are investigated in order to record and characterize their epileptic seizures. Before the start of the monitoring, scalp-EEG electrodes are fixed to the patient’s scalp. Afterward, these electrodes are connected to an amplifier and then converted from analog to digital signals, which are then displayed in a software program, allowing healthcare professionals to monitor brain activity in real-time, coupled with the video, electrocardiogram, and pulse oximetry signals. Patient monitoring lasts from 2 consecutive days up to two weeks. All the recording periods are available. Approval for retrospective data analysis with a waiver of informed consent due to the retrospective nature of the study was obtained from the local Ethical Committee of the University of Lausanne (study nr 2021-01419). The study report conforms to the STROBE statement for the report of observational cohort studies. All the methods are in accordance with institutional guidelines and regulations. Table 1 presents a summary of the employed datasets as well as the training duration.

**Table 1:** Summary of the employed EEG databases.

Patient	No. seizures	Recording duration	Avg. seizure length (s)	Training duration (h)
chb01	7	1d 17 h 33 min	63.14	34.63
chb02	3	1d 11 h 16 min	57.33	17.63
chb03	7	1d 14 h 2 min	57.43	31.69
chb04	4	6d 7 h 6 min	94.50	75.55
chb05	5	2d 15 h 12 min	111.60	47.40
chb06	10	2d 18 h 45 min	153.60	55.63
chb07	3	2d 19 h 5 min	108.30	33.54
chb08	5	1d 2 h 19 min	183.80	19.74
chb09	4	2d 19 h 52 min	69.00	33.93
chb10	7	2d 2 h 2 min	63.86	41.69
chb11	3	1d 9 h 45 min	268.70	16.88
chb12	40	21 h 41 min	36.63	19.27
chb13	12	11 h 0 min	44.00	9.43
chb14	8	1d 2 h 0 min	211.12	21.67
chb15	20	1d 15 h 1 min	99.60	36.02
chb16	10	17 h 1 min	8.62	13.61
chb17	3	20 h 1 min	97.67	10.01
chb18	6	1d 10 h 43 min	52.83	27.77
chb19	3	1d 4 h 53 min	78.67	14.44
chb20	8	1d 3 h 40 min	36.75	22.13
chb21	4	1d 8 h 23 min	49.75	21.58
chb22	3	1d 8 h 0 min	64.00	16.00
chb23	7	1d 2 h 36 min	60.57	13.3
Total	182	42d 1 h 59 min	–	633.55

P1	3	4d 10 h 59 min	488	53.49
P2	7	3d 20 h 32 min	64.86	77.10
P3	5	2d 21 h 58 min	117.20	52.47
P4	3	3d 20 h 13 min	117.98	46.11
P5	4	3d 18 h 23 min	35.25	60.25
P6	3	5d 19 h 23 min	118.67	69.69
Total	25	23d 1 h 28 min	–	359.11

#### Feature pre-processing

Starting from the raw data of the considered datasets, as a first step we extract DWT energy features^46^. DWT is widely used in preprocessing stages of machine learning algorithms, due to its ability to capture frequency and temporal features on a given signal. Unlike other signal transforms, such as Fourier transforms, DWT uses a series of filters called *Mother Wavelets* to decompose the input signal into a series of *approximation* and *detail* coefficients. These filters can be implemented as convolutions with pre-computed kernels, followed by downsampling by two. At each decomposition level, the time resolution is halved, and the frequency resolution is doubled, allowing for a more detailed representation of the original signal. This iterative process results in a series of *approximation* and *detail* coefficients that capture both the input signal’s low- and high-frequency components.

In bio-signal analysis, DWT effectively extracts features from physiological data due to its good trade-off between performance and signal-to-noise ratio^47^. We use a 4-level DWT with the Haar mother wavelet^42^ and temporal window lengths of 1 s, 2 s, 4 s and 8 s. We then calculate the energy of the detail coefficients at each level as a feature. When constructing the labeled data, we consider a window to be labeled as a seizure if the majority of samples (more than $50\%$) in that window are a seizure.

#### Gradient boosted trees

We base the classification task on Gradient Boosting^40^, which is a machine learning algorithm used to create predictive models. It builds a series of weak predictive models or weak learners, each slightly better than random guessing. These weak learners are combined to form a single robust model, which can make highly accurate predictions. XGBoost is a highly optimized and parallel version of the Gradient Boosted Tree algorithm which allows it to train the trees much faster. In this study, we use the XGBoost algorithm^44^ for the seizure detection.

#### Post-processing of labels

Epileptic seizures are expected to last multiple seconds when they occur. As in^48^, we use moving averages to smooth the predictions by applying a majority voting scheme. This operation has the effect of a low-pass filter, reducing false positives and eliminating fluctuations in the classifier output^41^. However, using a moving average filter also increases the latency of predictions, depending on the window size and the number of windows that are averaged. In the presented analysis, we average three successive classifications, meaning that a latency delay of one window size is obtained (for each time point, one window backward and one window forward are considered to perform the smoothing).

#### Model validation

In the training phase of the models under validation, a weighted loss function is utilized to heavily penalize false alarms. The loss function incorporated different weights for misclassification of *seizures* and *non-seizures*, similar to the approach used in^49^. We define the class weights as the inverse of the frequency of the occurrence of the two classes. In this configuration, the weight ratios are “balanced”.

Three methods for training and validating the models are also compared. Firstly, we closely look at a global model approach and a subject-specific approach. In the global model approach, we train a model on data from all the patients, while in the subject-specific, we train one model for each patient, with only data from that patient as training data. Additionally, for the subject-specific approach, we compare three approaches to validate the models. These approaches are based on EEG records (i.e., segmented portions of EEG data from an individual subject) and are as follows:

- Leave-One-Out Cross-Validation (LOOCV): we train on all records that have seizures in them except one and validate on the one left out.
- Walk-Forward Cross-Validation (WFCV)^50^: we keep a temporal coherency throughout the training and validation procedure. We never train on a record that happens after the validation record in time.
- Rolling Window Cross-Validation (RWCV)^51^: similar to the walk-forward approach except for the training records, since we do not train on records that happened very far behind the validation record.

While the first approach is commonly utilized in the literature for subject-specific analyses, the temporal dependencies inherent in the records motivate the exploration of the WFCV and RWCV.

Additionally, an extended analysis that accounts for the varying data volumes in each training loop of subject specific training is reported in the Supplementary Information.

### Artifact analysis methodology

Since neither the CHB-MIT dataset nor the PEDESITE dataset provide artifact labels, to enable the development of an EEG artifact detector, we make use of a subset of the TUH EEG Corpus, specifically the TUAR. The dataset includes 310 annotated EEG files from 213 patients. The TUAR dataset comprises 22 channels that have been separately annotated with 13 distinct labels, namely 12 artifact labels and 1 non-artifact label. Five sampling frequencies are available (namely, 250 Hz, 256 Hz, 400 Hz, 512 Hz, and 1000 Hz).

To to extract features from the dataset we require a constant sampling frequency. To this end, we refer to the results of^42^, where the sampling frequencies have been compared, and in the following, we focus only on the frequency that performed best in terms of accuracy (250 Hz). Thus, the dataset utilized in this paper comprises only the portion of the original dataset that was sampled at 250 Hz.

In this study, three approaches to classification are considered: Binary Classification (BC), Multilabel Classification (MC), and Multiclass-Multioutput Classification (MMC). In the following, the variable ${\Delta }T$ represents a generic time window, and $\Theta \left( \Delta T \right)$ denotes the label assigned to that window.

The BC approach labels a time window as an artifact $\Theta \left( \Delta T \right) =1$ if there is an artifact present on any of the channels. Conversely, if no channel is initially labeled with an artifact in that window, it is labeled as standard background EEG $\Theta \left( \Delta T \right) =0$.

In the MC approach, each channel is analyzed independently. Thus, an independent binary classification is performed on each channel. If the *i*-th channel was initially labeled with an artifact, the window is assigned a label of $\Theta _i \left( \Delta T \right) =1$. Conversely, if the *i*-th channel does not have an artifact in the considered time window, it is labeled as normal background EEG: $\Theta _i \left( \Delta T \right) =0$.

The MMC approach expands upon the MC approach by discriminating between the specific cause of the artifact among the 12 possible alternatives for each label of each channel. If the *i*-th channel was initially labeled with the artifact type *k* (where $k \in \left[ 1,12 \right]$), a label is assigned to that window for that channel as $\Theta _i \left( \Delta T \right) =k$. Similarly, if the *i*-th channel does not have an artifact in the considered time window, it is labeled as normal background EEG: $\Theta _i \left( \Delta T \right) =0$. Figure 1b shows a sketch of the workflow.

#### Feature extraction

Feature extraction is based on a combination of DWT and FFT. FFT is a computationally efficient method for calculating the frequency representation of time-domain signal values. However, the time information of the signal is lost after the transformation. FFT features have been shown to be effective for artifact detection^52^, and we use FFT to calculate the energy of the high-frequency parts of the signal (frequencies above 80 Hz), with the intuition that high energy in the high-frequency parts of the signal should be considered as not originating from the brain. For the DWT, instead, we use the same method as described in the feature extraction paragraph for seizures here above. Even though the FFT and DWT features can be used alone and give good metrics, relying on a combination of FFT and DWT features yields the best results.

#### Automated model optimization

After the feature extraction based on FFT and DWT, we utilize the TPOT^53^ for model selection and optimization. TPOT is an automated machine learning system that takes in features and labels and uses genetic programming to output the best model with cross-validated classification accuracy. By using TPOT, a comprehensive search is conducted over a wide range of machine learning models. Among the available AutoML frameworks^54–56^, we chose to use TPOT by virtue of its rapid development time.

#### Performance metrics

We evaluate the models obtained via TPOT according to classification accuracy, i.e., the ratio between correctly classified trials and the total number of trials in the validation set. Since the TUAR dataset we use in this paper is imbalanced, we also consider the F1 score. For the **MMC** case, we report weighted F1 scores considering each class’s support.

### Artifact and seizure combination methodology

Starting from the developed seizure and artifact detectors (described here above), we target combining these approaches into an integrated framework. However, a significant challenge is given by the lack of datasets labeled for both seizures and artifacts. To address this challenge, we propose to leverage statistical analysis to combine two distinct datasets. Moreover, to further validate the framework, we also verify the results on an alternative dataset (Temple University Event Corpus (TUEV)) labeled for artifacts and epileptic discharges.

#### Combination of two datasets

We explore three approaches to combine a seizure dataset (CHB-MIT) with an artifact dataset (TUAR). To make the section more readable, in the following, we will refer to the artifact detection model as **Classifier A** and to the seizure detection model as **Classifier S**. The three approaches are based on different normalization techniques and are as follows:

- *raw data (no normalization):* we train **Classifier A** on the artifact training data, and then we train **Classifier S** on the seizure training data.
- *min–max normalization:* we train **Classifier A** on the artifact training data that have been Min–Max scaled from 0 to 1, and then we train **Classifier S** on the seizure training data that has also been scaled from 0 to 1.
- *z-score normalization:* we train **Classifier A** on the artifact training data that have been standard scaled (removed the mean and scaled to unit variance), and then we train **Classifier S** on the seizure training data that also has been standard scaled.

Then we analyze the effect of passing data from the artifact dataset (**A**) through **Classifier S** and feeding data from the seizure dataset (**S**) through **Classifier A**. Such analysis aims at understanding:

- Are artifacts being classified as seizures? Is there a difference between the artifacts that are not detected by the artifact detector and those that are detected?
- Are seizures being classified as artifacts, therefore using an artifact detector results in lower sensitivity scores in the overall workflow?

#### Epileptic discharges dataset

The main limitation of the analyses above is the mixing of heterogeneous datasets. In order to further validate the effectiveness of our proposed approach for combining artifact and seizure detection using a unique dataset (i.e., not mixing heterogeneous sources), we utilized another subset of the TUH EEG Corpus named the TUEV that contains annotations of EEG segments as one of six classes: (1) spike and sharp wave, (2) generalized periodic epileptiform discharges, (3) periodic lateralized epileptiform discharges, (4) eye movement, (5) artifact and (6) background EEG. The TUEV dataset is considered as a representative example of dataset with labels for artifacts and epileptic activity (interictal epileptic discharges), despite not containing epileptic seizures. Given the nature of the TUEV dataset, which labels epileptic discharges rather than seizures and only includes 1-s epochs, we extracted two separate sub-datasets, one consisting of background EEG and the combination of artifacts and eye movements, and the other consisting of background EEG and epileptic discharges. We train an artifact detector on the first dataset and a discharge detector on the second.

We conclude the analysis by repeating the process of passing data from the artifact dataset into the discharge detector and vice versa, to evaluate the potential impact of artifacts on epileptic discharges detection sensitivity. In this case, no additional scaling is required to obtain reliable results as the data originated from the same dataset. The same methodology could be applied for combined artifacts and seizure detection, if a single dataset with labels for both were available.

### Embedded implementation methodology

#### GAP9 platform

In our previous studies^41,42^, the seizure detection and artifact detection frameworks have been implemented and optimized on the BioWolf wearable ExG device^57^, which features efficient on-board processing capability thanks to the PULP Mr. Wolf chip. In the present study, we focus on the optimization of the models for the GAP9 device which is a new commercialized generation of a more energy-efficient PULP processor^58^. We target the new GAP9 processor since it offers the best trade-off energy efficiency and performance in the target power envelope of milliwatts^59^ requested for wearable battery-operated devices. In fact, the GAP9 outperforms by at least one order of magnitude the conventional single-core low-power processors, such as ARM CORTEX M4 device^10^, with a comparable power budget.

We select this platform also by virtue of its parallel computation capabilities. In fact, GAP9 is a low-power parallel microcontroller with ten cores based on the RISC-V RV32IMF Instruction Set Architecture, with custom Xpulp extensions for digital signal processing^58^. The ten cores of GAP9 are split into Fabric Controller (1) and Cluster Cores (9). GAP9 also features 128 kB L1 memory and 1.5 MB RAM, allowing relatively large models to be implemented. The cluster cores can run up to 370 MHz and share 4 Floating-Point Units, supporting operations in bfloat, FP16, FP32, and (for some instructions) also FP64.

#### Feature extraction implementation

The FFT implementation we developed on GAP9 utilizes the conjugate symmetry property of real-valued FFT to optimize computational efficiency. By implementing a complex FFT on half of the signal, the real-valued component is extracted from the output, resulting in a significant reduction in computation as compared to performing a full real-valued FFT on the entire signal. Additionally, the implementation uses a mixed-radix complex FFT approach to optimize processor utilization further. As for the DWT implementation, it iteratively passes through low-pass and high-pass filters. Given the focus on temporal channels, which are four in total, the computation of features is split such that four cores are allocated to computing the FFT, while another set of four cores are allocated to the DWT. This results in two cores per channel dedicated to feature extraction.

#### Artifact model pruning

The optimized artifact detection models generated by TPOT are mainly Extra Trees, i.e., an ensemble of Decision Trees (DTs)^60^. The obtained DTs can hardly fit on an embedded platform (they feature millions of threshold values). Therefore, in^42^ we applied pruning on the DTs with a MCCP algorithm^61^ in order to prune the DTs down such that the tree would fit on the L1/L2 memory of the targeted processor.

Additionally, since Gradient Boosted models (and in particular, XGBoost) proved to perform better than other tree-based models for seizure classification, we also explore their applicability. To this end, we use the TPOT-optimized hyperparameters and prune the trees for fitting the model on the L1/L2 memory of GAP9.

#### Tree ensemble implementation

The Gradient Boosting Classifier is a machine-learning technique that utilizes an ensemble of regression trees. Each tree in the ensemble is constructed by repeatedly splitting internal nodes based on specific features and threshold values. The implementation of this model is inspired by tree-based models presented in previous research^42^.

To extract the necessary information for each tree, three arrays are utilized: the feature array, which indicates the feature to be compared to the threshold value; the index array, which specifies the next internal node or leaf node to be traversed; and the threshold array, which contains the threshold values used for comparisons. The feature array is represented with 8 bits, while the index array is represented with 16 bits and the threshold array is represented with 32 bits. Each node in the regression tree, therefore, requires 7 bytes of memory. These arrays are then stored on L2 memory and are processed by the GAP9 cluster cores.

To optimize computational efficiency, the number of trees in the ensemble is adapted to be divisible by the number of cores, and the output value is obtained by summing the individual predictions of each tree.

#### Energy measurements

The GAP9 Evaluation Kit is utilized for the purpose of energy measurements. The evaluation kit is supplied with 5 V and an onboard DC-DC converter generates the 1.8 V supply for the system-on-chip. The 0.65 V supply for the processor is generated through an on-chip DC-DC converter and is made accessible for power measurements outside of the system-on-chip. The evaluation kit has dedicated power consumption test points for the 0.65 V domain, through which the current and energy consumption are determined by calculating the voltage drop across a 0.5 $\Omega$ resistor that is connected to the processor’s supply input. This calculation is performed in accordance with Ohm’s Law. The power measurements are executed utilizing a Keysight InfiniiVision MSO-X 2024A oscilloscope. Two passive probes are attached to the GAP9 test points with the aim of measuring the voltage drop, with their grounds connected together. A third probe is connected to a GPIO pin of the evaluation board for the purpose of synchronizing the measurements across distinct runs, with its ground connected to the board’s ground. The probes are bandwidth-limited to 20 MHz to minimize noise and ensure a clean signal. The performance numbers are calculated as the average of ten consecutive measurements. To compensate for the losses incurred by the on-chip DC-DC converter, which boasts a $90\%$ efficiency, an additional $10\%$ is added to the measured processor power numbers.

## Results and discussion

### Seizure analysis results

Table 2 compares our work to the existing literature on low channel count systems that aim at minimizing the false alarms. Our approach excels in FP/h, the most important metric for patient acceptance, while being competitive in sensitivity. While further improvement is still needed to reach the needed level of acceptance for all patients (i.e., at most 1 FP/day), most subjects exhibit no false positives at all, as detailed in the following.

**Table 2:** Summary of scalp EEG-based seizure detection processing SoA for low channel count.

Work	Dataset	Length	Window	Subjects	Channels	Algorithm	Sens (%)	Spec (%)	FP/h (%)
^19^	CHB-MIT	944 h	4 s	23	2	RF	96.6	92.2	–
^62^	Uni. Hospital Leuven	5284 h	2 s	54	4	SVM	63.4	–	0.9
^63^	CHB-MIT	464 h	8 s	8	4	Transformer	65.5	99.9	0.8
^18^	EPILEPSIAE	4603 h	3 s	29	3	CNN	87.0	95.7	52.0
^20^	CHB-MIT	944 h	1 s	23	5	RF	99.8	99.8	4.3
This work	CHB-MIT	944 h	2 s–8 s	23	4	XGBoost	65.3	**99.9**	**0**.**65**
This work	PEDESITE	591 h	2 s–8 s	6	4	XGBoost	57.3	**99.9**	**0**.**51**

Best values are in bold.

#### CHB-MIT dataset

**Figure 2:**
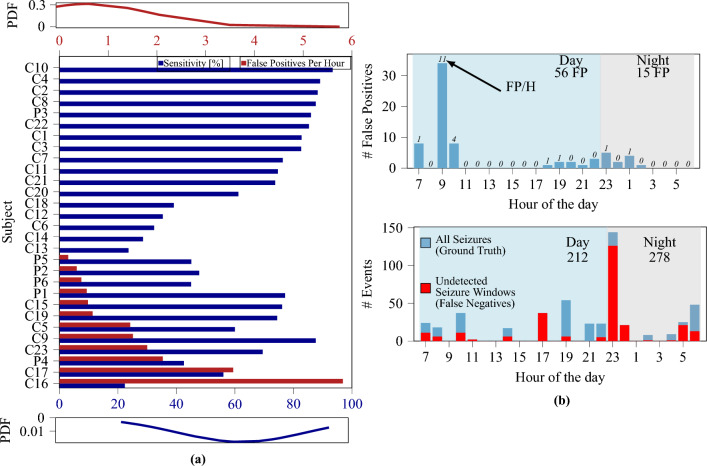
(**a**) Sensitivity and FP/h histogram of all considered patients. Labels “C” and “P” indicate patients of the CHB-MIT and PEDESITE datasets, respectively. Approximately $60\%$ of patients exhibited zero false alarms. Top/bottom insets: probability density function (PDF) for the FP/h and sensitivity metrics. (**b**) Distribution of FP (left) and all/missed seizures (right) over hours of the day. Dataset: PEDESITE.

Similarly as done before^41^, we use a global model and optimize the window length to maximize sensitivity and specificity. Compared to before^41^, where only a subset of patients was considered, we use the entire CHB-MIT dataset, making the classification more challenging. As previously reported^41^, the utilization of larger temporal windows improves specificity and sensitivity. However, the achieved sensitivity is notably lower (65%) compared to the result achieved on a limited number of subjects (84.3%). These results further demonstrate the necessity for subject-specific models, and Table 3 summarizes the performance of three validation methods in a subject-specific training and validation approach. The results (which are averaged across all patients) indicate that the LOOCV method yields the highest performance. Additionally, while a temporal window size of 4 s yielded the best results when averaging across all patients, the optimal window size also appeared to be patient-specific. When considering a variable window size (optimized on each patient) the performance scores further improved, achieving a sensitivity of 65% and an average false positive rate per hour of 0.65, with 16 out of 23 subjects resulting in zero false positives (see Fig. 2a).

**Table 3:** Comparison of the three validation methods (LOOCV, WFCV, RWCV) on the whole CHB-MIT dataset, and the best method (LOOCV) on the whole PEDESITE dataset. The range of the $95\%$ confidence interval is indicated below each number. Model:XGBoost, subject-specific.

		LOOCV (CHB-MIT)	WFCV (CHB-MIT)	RWCV (CHB-MIT)	LOOCV (PEDESITE)
1 s Window	Sens (%)	61.95 ± 10.67	58.57 ± 11.44	59.12 ± 11.01	53.25 ± 20.04
	Spec (%)	99.87 ± 0.10	99.90 ± 0.07	99.89 ± 0.07	99.89 ± 0.10
	FP/h	4.67 ± 3.76	3.60 ± 2.41	3.65 ± 2.52	4.08 ± 3.71
4 s Window	Sens (%)	64.09 ± 10.88	60.70 ± 12.05	61.3 ± 11.05	57.73 ± 17.29
	Spec (%)	99.91 ± 0.08	99.81 ± 0.19	99.81 ± 0.12	99.91 ± 0.07
	FP/h	0.83 ± 0.78	1.73 ± 1.67	1.73 ± 1.08	0.80 ± 0.60
8 s Window	Sens (%)	56.21 ± 12.49	54.10 ± 12.17	54.15 ± 12.30	56.80 ± 15.54
	Spec (%)	99.69 ± 0.31	99.61 ± 0.30	99.62 ± 0.30	99.86 ± 0.14
	FP/h	1.40 ± 1.41	1.74 ± 1.34	1.65 ± 1.35	0.62 ± 0.61
Variable window size	Sens (%)	**65.27** ± 9.59	61.25 ± 12.38	62.3 ± 11.00	**57.26** ± 15.30
	Spec (%)	**99.90** ± 0.09	99.82 ± 0.18	99.82 ± 0.14	**99.89** ± 0.10
	FP/h	**0.65** ± 0.58	1.09 ± 0.99	1.09 ± 0.63	**0.51** ± 0.44

Best values are in bold.

#### PEDESITE dataset

**Figure 3:**
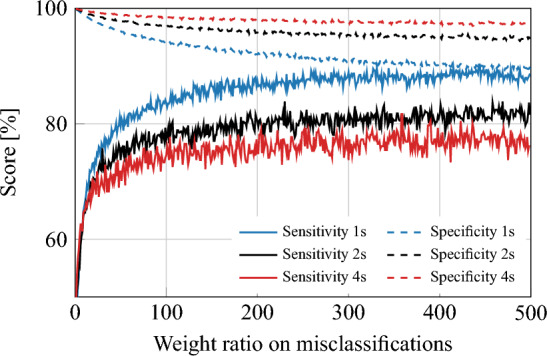
Sensitivity and specificity plot for the global model approach on the PEDESITE dataset, illustrating the impact of the weighting ratio and the choice of window length (1, 2, or 4 s) on the performance of the model. As the weight ratio increases, the model’s sensitivity improves, but at the cost of decreased specificity. Conversely, as the window length increases, the model’s specificity improves, but at the cost of reduced sensitivity. A weight of 1 corresponds to the no-weighting case.

A global model analysis is performed to confirm the understanding obtained from the analysis on the CHB-MIT dataset. The trends in the PEDESITE dataset in the global model approach were consistent with those in the CHB-MIT dataset. Specifically, the sensitivity and specificity of the model increased as the temporal window size increased. However, as illustrated in Fig. 3, the specificity is low and therefore resulting in a false positive rate per hour (FP/h) that is still too high for practical usage. The LOOCV method was then applied for validating subject-specific models on the PEDESITE dataset. We followed the same training procedures as reported for the CHB-MIT dataset. Table 3 presents the results of varying the temporal window size. The classification results, in terms of specificity, sensitivity, and false positive rate per hour (FP/h), appear to reach an optimal balance at a temporal window length of 4 s.

Figure 2a shows the sensitivity and FP/h rate for all considered individual patients. These results demonstrate that the proposed method could be used in a selected population where it provides high sensitivity or being the first step of a solution aimed at progressively learning from the individual patient. Figure 2b shows the distribution of the FP (top) and seizures (bottom) during the hours of the day. Most FP occur during wake, and the peak at 9:00 might correspond to bathroom time (shower, tooth brushing, etc.) which causes artifacts. FP during the night (most annoying for patients) are minimal. Hence, the proposed solution appears as already feasible for real-life use during sleep in view of the very low FP/h rate (see Fig. 2).

### Artifact analysis results

**Figure 4:**
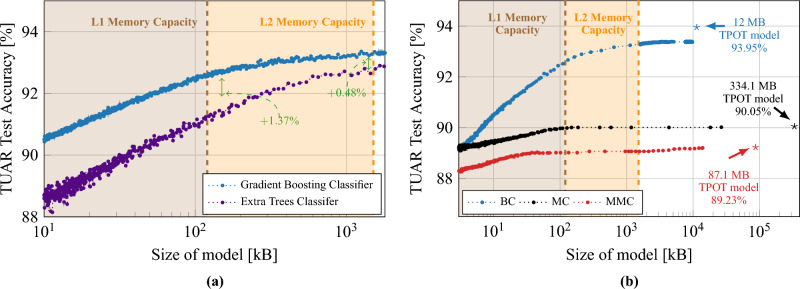
Comparative analysis of artifact detection, in both sub-figures the shaded areas (brown and orange) represent the L1 and L2 memory capacity of GAP9. (**a**) Compares artifact detection accuracy of classifiers against model size, illustrating superior resistance and performance of a Gradient Boosting Classifier versus an Extra Trees Classifier optimized by TPOT. (**b**) Extends to Gradient Boosted Trees’ accuracy for three labeling methods (BC, MC, MMC) when implemented on the GAP9 processor. Star symbols denote TPOT-optimized models’ F1 scores (0.84, 0.60, 0.87 for BC, MC, MMC, respectively).

Figure 4a summarizes the artifact detection accuracy when using TPOT, demonstrating the feasibility of accurate artifact classification via minimal-montage EEG setups. The study focuses on the Temporal Central Parasagittal (TCP) average referenced montage^64^, particularly on the four temporal channels (F7–T3, T3–T5, F8–T4, T4–T6) as specified by the 10–20 international system notation. Gradient Boosting appears as superior compared to Extra Trees, with an increased accuracy of $1.4\%$ for L1-sized models.

We optimized Gradient Boosted Classifiers for use on the GAP9 processor and limit the memory usage to the available L2 memory of 1.5 MB. Initially, we limited the number of DTs in the ensemble to be a multiple of the number of cores on GAP9, to optimize parallelization on the 9-cores cluster. We then use the MCCP algorithm to eliminate the weakest links in the DTs. Figure 4b illustrates the outcome of this optimization process. We apply these limiting and pruning operations for all three cases (BC, MC, MMC) of the 250 Hz dataset. Further optimization is carried out using the MCCP algorithm, progressively increasing the complexity parameter that controls the degree of pruning. For the **BC** case, we reach a $93.3\%$ accuracy when the model’s size matches the size of the L2 memory (1.5 MB), which corresponds to a decrease of only $0.65\%$ from the optimal model identified by using TPOT. For the **MC** and **MMC** cases, we can prune the DTs more aggressively with minimal loss in accuracy, until it fits the size of the L1 memory. This minimal loss of accuracy with pruning confirms that embedded implementations of models with low memory usage can compete with state-of-the-art models.

### Artifact and seizure combination results

Table 4 compares our work with the existing approaches, with a focus on the false positive improvement, i.e., how much the method improves the number of false positives, when accounting for artifacts in EEG data.

**Table 4:** Summary of artifact and seizure combination works.

Work	Dataset	Approach	FP improvement (%)
^38^	CHB-MIT synthetic artifacts	Cleaning	80
^39^	Freiburg EEG recorded artifacts	Cleaning	49
This work	CHB-MIT/PEDESITE TUH-EEG	Detection	**96**

Best values are in bold.

#### Artifact data fed to Seizure detector

**Figure 5:**
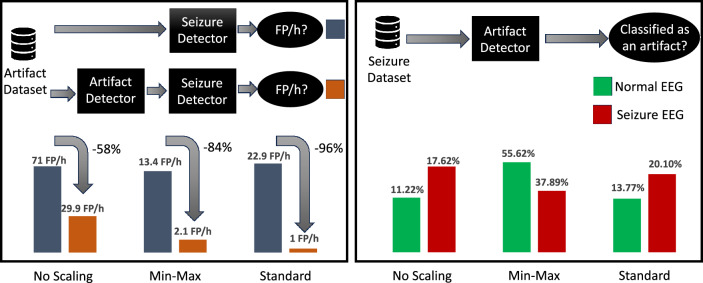
Left: comparison of the difference in FP/h when passing artifact data through the seizure detector with and without an artifact detector in front. Right: the probability of classifying normal EEG and seizure EEG as an artifact when feeding seizure data into the artifact detector (combined datasets approach).

We evaluate three methods for combining the seizure and artifact datasets. The first method did not involve any scaling and is expected to be less effective due to differences in data acquisition and signal amplitudes between the two datasets. Indeed, when normal EEG data (i.e., data without artifacts) is passed through the seizure detection model (Classifier S) it has a 50/50 chance of giving a false positive (i.e, classifying the normal EEG as a seizure) – an unacceptably low level of performance. Figure 5 showcases the improvement of including an artifact detector (Classifier A) before passing data through Classifier S. Indeed, the improvement ranged from $58\%$ to $96\%$ with the standard scaling method having the best performance. Additionally, normal EEG data have only a small chance ($0.04\%$) of being incorrectly classified as seizures using this standard scaling method. These results quantitatively confirm that using an artifact detector before a seizure detection model allows to significantly reduce false alarms.

#### Seizure data fed to artifact detector

A question left unanswered is: does the artifact detector affect the sensitivity of the seizure detector? In other words, does the artifact detector prevent some seizures from being classified and detected? Figure 5 shows that when utilizing a standard scaling method for data pre-processing, approximately $20\%$ seizure EEG data are classified as an artifact. Upon further analysis, we notice that the majority of these instances occur within the initial stages of the seizure, with the remaining artifact misclassifications being scattered temporally and not bound to impact the overall sensitivity of the system significantly. On an aggregate level across all subjects, an average of 1.2 segments per seizure event is misclassified as artifacts, translating to an added latency of approximately 4.8 s in detection. Furthermore, roughly $13.8\%$ of normal EEG (i.e., no seizures present) are classified as an artifact. Going by the results in the artifact detection, it is evident that the percentage of normal EEG data classified as artifacts is an overestimation, this most likely is a result of the difficulty of mixing two datasets. Therefore, we conclude that $20\%$ of seizure data being classified as an artifact is also an overestimation and will be lower in a real-life example and, therefore, will affect sensitivity less. These findings suggest that, while the incorporation of artifact detection may result in an increase in latency in seizure prediction, it also makes the predictor less prone to EEG artifacts. In addition, we notice that seizure data (from the seizure dataset) misclassified as artifacts (by the artifact detector, trained with the artifact dataset) tend to: either group at the beginning of a seizure, or appear as scattered without exhibiting a specific pattern. Thanks to the smoothing approach of the seizure detector, the misclassifications that group at the beginning of seizure events result in an increased latency of detection, whereas scattered misclassifications get smoothed out without affecting sensitivity. Hence, sensitivity appears to be affected only by misclassifications falling outside of the two groups above, which account for less than 5% of the total seizure windows.

#### Epileptic discharges dataset

Similar trends are present for the TUEV dataset. Specifically, when passing artifact data into the artifact-discharges combined framework, as the number of false positives reduces by $96\%$ or more specifically from 6362 to 290, this is an FP/h decrease of 69.0 FP/h $\rightarrow 3.1$ FP/h. Additionally, when feeding discharge data into the artifact-discharges combined framework, approximately $30\%$ of the discharges are initially classified as artifacts, which is consistent with the results obtained in the previous dataset combination analysis. However, due to the limited nature of the labeled data in the TUEV dataset, it is difficult to accurately determine the impact of this misclassification on the overall system sensitivity in a real-life scenario.

### Embedded implementation results

**Figure 6:**
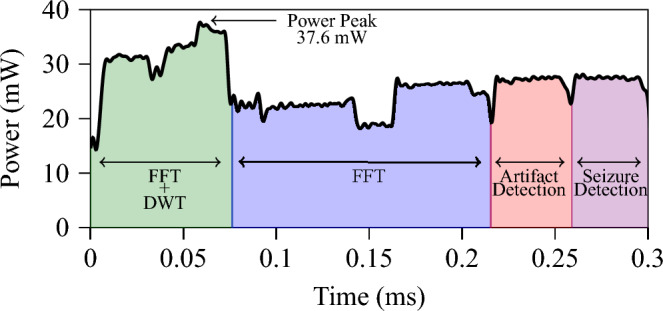
Power trace of the whole framework implemented on GAP9.

We implement the whole framework (i.e., feature extraction, artifact detection and seizure detection) on GAP9. We chose a 4 s window for implementation as it showcased the best average performance in the seizure detection case. The power trace of the 4 s window can be seen in Fig. 6. The feature extraction takes roughly two times as long as the classification stage, and the total time for feature extraction and classification of both artifacts and seizures is remarkably performed in approximately 0.3 ms. Such a result is achieved while consuming a max peak power of 37.6 mW and an average power envelope of only 26.1 mW, with total energy consumption as low as 7.93 $\upmu$J. Assuming to integrate the proposed GAP9 implementation with an SoA commercial analog front end for biosignal acquisition (such as the Texas Instruments ADS1298^57^, which requires 0.75 mW per channel) and considering a battery of 300 mAh, the proposed approach ensures approximately 300 h of continuous data acquisition and classification at 4 s intervals, allowing for multi-day functionality.

## Conclusion

A framework for seizure and artifact detection was developed and analyzed. The proposed seizure detection algorithm is based on Gradient Boosted Trees (XGBoost) and has been evaluated on the CHB-MIT dataset and on a private dataset. An average sensitivity of $65.27\%$ and $57.26\%$ was achieved on the two datasets, respectively, also guaranteeing less than 0.58 FP/h on average, with most patients ($60\%$) experiencing no false positives at all, thereby showcasing the potential for implementation in real life. An artifact detection framework has also been proposed and tested on the TUAR dataset, evaluating the performance of pruned Gradient Boosted Trees and Extra Trees Classifier and achieving $93.95\%$ accuracy on the TUAR dataset. Finally, the combination of seizure and artifact detection has been explored. We demonstrated the importance of incorporating an artifact detector in conjunction with a seizure detection framework to reduce the number of false alarms. In fact, using the artifact detector allowed for a $96\%$ reduction in FP/h.

The proposed framework also allows for multi-day continuous operation when executed on an ultra-low-power device. In fact, by optimizing the implementation of the framework for a PULP platform, we demonstrated a power envelope of 26.1 mW and energy usage as low as 7.93 $\upmu$J per window feature extraction and classification.

Our algorithms were primarily developed for standard EEGs with traditional electrodes. As ambulatory EEG devices diversify, further validation will be needed for different devices. Moreover, while our current methodology emphasizes artifact rejection for clarity in EEG-based detection, we cannot ignore the potential diagnostic value of certain artifacts, such as changes in Electrocardiogram (ECG) rate or muscle movements, in seizure identification. Future research should not only adapt our methods for wearable and alternative electrode arrays but also contemplate AI-driven approaches that harness these informative artifacts. Such strategies, which might bypass predefined feature dependencies, would benefit from expansive, well-annotated databases, highlighting an intriguing direction for subsequent investigations. In fact, artifacts generated by seizures might also provide different patterns compared to the physiological ones, hence offering additional opportunities for new characterization strategies.

Another field of exploration for further research is integrating the proposed methods with other biosignal data modalities, such as wrist-worn wearable IoT devices. Such integration could enrich the detection framework and bolster its specificity, potentially further minimizing false positives (especially in the context of real-life activities, as compared to analyses of patients staying quiet in hospital beds^65^).

Finally, approaches that allow minimizing the amount of data needed for subject-specific training also need to be explored. While our methodology aligns well with offline EEG analysis in home monitoring settings using traditional systems like the 10–20, its main intended purpose is to enable versatility for online analysis in wearable platforms, especially in real-time applications in diverse wearable EEG monitoring scenarios.

### Supplementary Information

Supplementary Information.

## Data Availability

The artifact data used in this paper is released by the Temple University Hospital through the following website at https://isip.piconepress.com/projects/tuh_eeg/. The seizure data used in this work is from a public database (CHB-MIT scalp EEG database), which could be accessed and downloaded via https://physionet.org/content/chbmit/1.0.0/. The dataset generated during and/or analysed during the current study are available from the corresponding author on reasonable request.
