# Supplementary material for: Minimizing artifact-induced false-alarms for seizure detection in wearable EEG devices with gradient-boosted tree classifiers
Source: Sci Rep. 2024 Feb 5;14:2980. doi: 10.1038/s41598-024-52551-0 (PMC10844293; doi:10.1038/s41598-024-52551-0)
Supplement: Supplementary file 1 — Supplementary Information. [file 41598_2024_52551_MOESM1_ESM.pdf]

Supplementary Information

Minimizing Artifact-induced False-Alarms for Seizure Detection in Wearable EEG Devices with Gradient-Boosted Tree Classifiers

Thorir Mar Ingolfsson[1,*], Simone Benatti[2,3], Xiaying Wang[1], Adriano Bernini[4], Pauline Ducouret[4], Philippe Ryvlin[4], Sandor Beniczky[5,6], Luca Benini[1,2], and Andrea Cossettini[1]

[1]ETH Zürich,D-ITET, Zürich, 8092, Switzerland
[2]University of Bologna, Bologna, 40126, Italy
[3]University of Modena and Reggio Emilia, Reggio Emilia, 41121, Italy
[4]University Hospital of Lausanne (CHUV), Lausanne, 1011, Switzerland
[5]Aarhus University Hospital, Aarhus, 8200, Denmark
[6]Danish Epilepsy Centre (Filadelfia), Dianalund, 4293, Denmark
[*]thoriri@iis.ee.ethz.ch

Data required for a Subject Specific Classifier

Subject-specific classification faces the main challenge of the scarce amount of data that can be used for training. In this context, we extend the investigation reported in the main text of the manuscript by addressing the following question: ***What is the minimal dataset size required for the training of a robust subject-specific classifier?***

The approach presented in the main text of the manuscript (see Sect. Methods) is based on Leave-One-Out Cross-Validation (LOOCV) experiments, where:

- the model is trained on all but one seizure file and then validated on the excluded file;
- this process is repeated for all seizure events (each seizure file being left out once);
- the results are averaged over all iterations.

In this Supplementary Information, we extend these analyses in order to account for the varying data volumes in each training loop. Specifically, we present ablation experiments, directly comparing to the results with the full dataset size from the original experiments (see Sect. Methods of the manuscript) and revealing the impact of the amount of training data on the different performance metrics.

Methods

Experiments are conducted as follows. For each subject, data are split into distinct seizure events files (the total number of seizure events N_s for each subject is indicated in Table 1 of the manuscript). Furthermore, each seizure event file can have more than one seizure, thereby the number of seizure event files N_F is smaller than N_s. Subsequently:

- We initially train a classifier on one single seizure file and perform validation on the remaining $N_F - 1$ ones. The process is repeated by executing all unique permutations of such training over the single seizure event files.
- In progressive iterations, we include one additional seizure file and validate on the remaining ones, exploring all possible unique permutations.
- The last iteration corresponds to the case of training on $N_F - 1$ files and validating on the remaining one (exploring all possible unique permutations).

For each iteration, the number of explored permutations corresponds to $\binom{N_F}{N_t}$, where N_F and N_t are the total number of seizure files and the number of seizure files used in training. Hence, the total number of explorations for the complete study of a single subject (with N_F seizure files) corresponds to: $\sum_{k=1}^{N_F-1}\binom{N_F}{k}$.

For example, for a subject with $N_F = 7$ seizure files, we:

- Train on one event and validate on the remaining six (7 runs in total).
- Train on two events and validate on the remaining five (21 runs in total).
- Continue this pattern up to training on six events and validating on one.

for a total of 126 runs. Subjects with a greater number of files require a progressively larger number of runs to cover the whole design space (for instance, Subject 15, who features 14 distinct event files, required 16,382 runs). We also repeated the experiments for each window size (1, 2, 4, and 8 seconds). As such, the proposed experiments have a combinatorial complexity.

For each run, we calculate the average of the performance metrics — specificity, sensitivity, and false positive rate. We also record the mean training duration in hours. This enabled us to analyze the classifier's performance relative to the amount of training data used.

Noticing the variability in the amount of training data in each patient, we perform linear interpolations to estimate performance metrics for standard hour ranges (5 to 65 hours of EEG signals). Interpolations are based on the available data points for each patient. For instance, if a patient had data points at 5 and 15 hours, we linearly interpolate the performance at the 10-hour mark. Similarly, if a patient does not have enough hours of data, they are omitted from further averaging as indicated on the x-axis in Figure S1.

Furthermore, we determine the optimal window size for each patient by comparing the interpolated results across different window sizes within each hour range. An average plot is then calculated by iterating through all the hour ranges and patients, providing a comprehensive view of the optimal window sizes for seizure detection.

Finally, beyond displaying average data across patients, to account for the great inter-subject variability that significantly affects the averages, especially when a very small number of subjects is considered, we also report numbers on a per-subject basis.

Results

Aggregated performance analysis

Figure S1 presents the mean performance metrics aggregated across all participants. This illustration corroborates the anticipated hypothesis: a direct correlation between the enhancement in classifier efficiency and the augmentation of training data volume. Detailed statistical representations, including the mean values corresponding to each duration of the training and the associated 95% confidence intervals, are tabulated in Table S1.

Further analysis of Figure S1 reveals a notable increment in the false positives per hour (FP/h) metric between 20 and 25 hours and 35 and 40 hours. These increments are attributed to the drop in the number of patients featuring such a high number of recording hours. More specifically, the drop results from the exclusion of the two high-performing subjects C1 and C15 (see Fig. 2 of the manuscript), who do not have more than 40 hours of data, thereby negatively affecting the 40-hour data point. The same reasoning can be applied to the increase of FP/h from 20 (17 averaged patients) to 25 (10 averaged patients) hours. Concurrently, subjects C5 and C9, characterized by elevated FP/h rates, are retained (as illustrated also in Figure 2 of the manuscript, they present a less efficient performance).

Similarly, a decline in sensitivity is observed around the 45-hour mark, attributed to the loss of two proficient subjects (C7 and C10) while retaining a challenging subject, C6. This subject is also identified as one of the lower performers in the main manuscript's Figure 2. This pattern persists in subsequent training durations, marked by the attrition of higher-performing subjects and the persistence of those presenting greater challenges.

Per-subject analysis

In Figure S2, we delineate the individual patient variability in classifier metrics as juxtaposed against their respective asymptotic baselines. Specifically, for each performance metric M calculated at each training-hour range $M(h)$, we plot a "saturation index" $S(M,h)$, defined as $S(M,h) = M(h)/M(h=\infty)$, where $M(h=\infty)$ represents the value at the last data point (at the highest number of training hours). Such quantity enables to better visualize and identify at which training-hour range the performance metric converges to the final asymptotic value. Upon examination, a general trend of metric saturation is discernible for most subjects around the 30-hour training threshold. However, while a general saturation trend applies across all subjects, the quantitative minimum number of hours of data

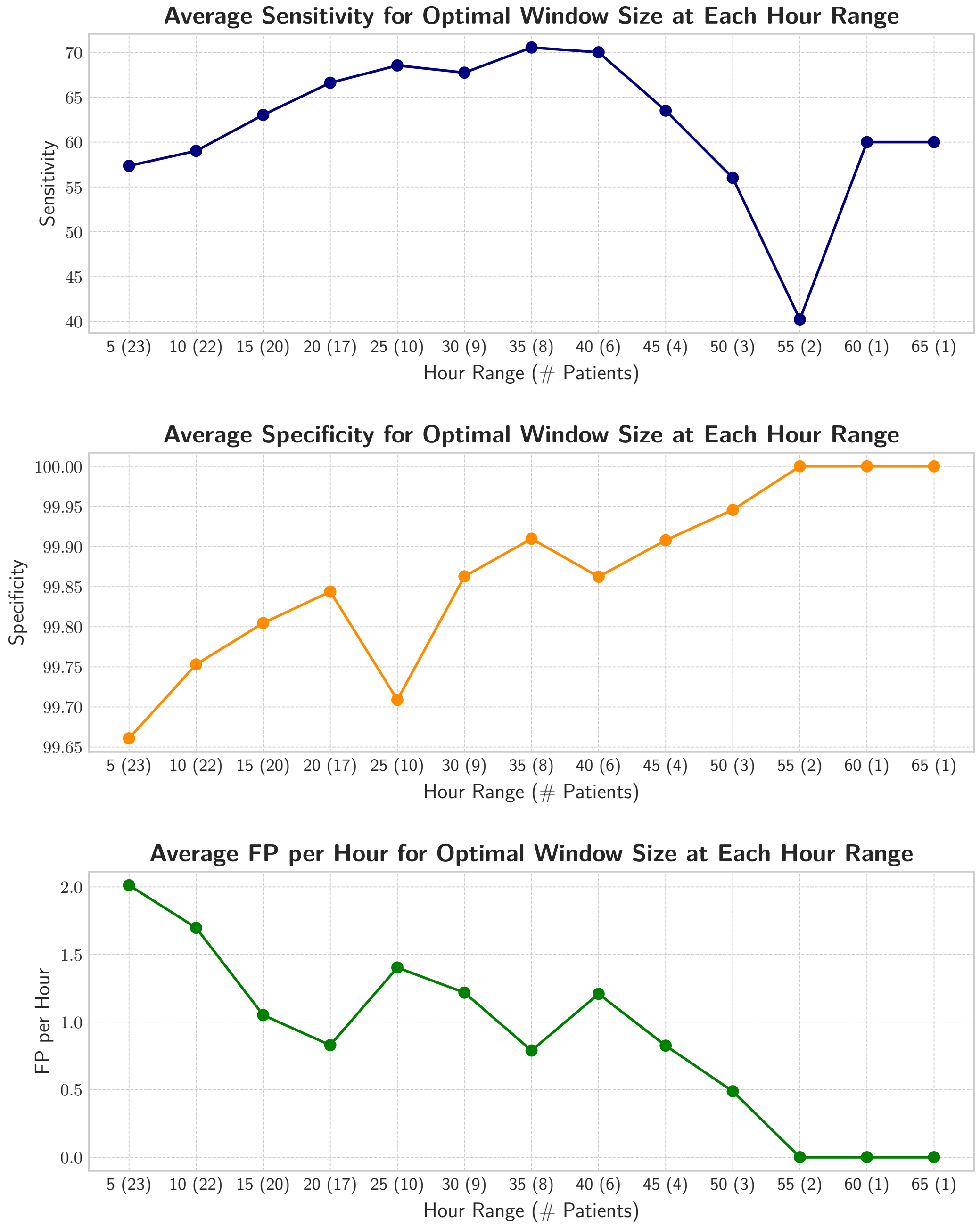

Figure S1. Averaged classifier performance metrics over diverse training durations. The x-axis on the plots also indicates the number of patients used for averaging within parenthesis, and the number decreases as fewer patients have long hours of recordings. Dataset: CHB-MIT

needed for training differs across subjects, also in agreement with the discussion articulated in the manuscript (Sect. Results and Discussion). In short, this observation underscores the potential utility of the proposed methodology within a select patient cohort characterized by high sensitivity and minimal false positives per hour. Alternatively, it suggests the feasibility of this approach as an initial step in a progressively adaptive learning framework tailored to individual patient profiles.

Extending beyond the fundamental premise that increased hours of personalized training improve performance metrics, the reported results reveal how the specificity of our model tends to saturate beyond a certain threshold of training data (50 hours). This saturation suggests a limit to the benefits of additional data in enhancing the model's specificity, implying that further data collection may yield diminishing returns beyond this point. As regards the sensitivity, the same conclusions can be derives observing that the model continues to show improvement with more data until 55 hours of training. These findings highlight the nuanced balance between data volume and model performance, especially in the context of the practical challenges in personalized training for real-life applications. Thus, the development of strategies to make personalized training more efficient and feasible for a real-world setting appears of paramount importance and will be explored as future work.

Table S1. Comparison across different durations of training hours for variable window size. The 95% confidence interval range is indicated below each number; the range has been cut where applicable so numbers are within a logical range.

Hours of training data	Sensitivity [%]	Specificity [%]	FP/h	# Patients
5	57.36<br>(47.54, 67.17)	99.66<br>(99.40, 99.92)	2.01<br>(0.79, 3.24)	23
10	59.02<br>(49.42, 68.62)	99.75<br>(99.59, 99.92)	1.70<br>(0.70, 2.70)	22
15	63.03<br>(54.90, 71.16)	99.80<br>(99.61, 99.99)	1.05<br>(0.21, 1.89)	20
20	66.62<br>(57.69, 75.54)	99.84<br>(99.63, 100)	0.83<br>(0, 1.78)	17
25	68.55<br>(58.23, 78.82)	99.71<br>(99.32, 100)	1.40<br>(0, 3.12)	10
30	67.74<br>(51.04, 84.44)	99.86<br>(99.66, 100)	1.22<br>(0, 3.02)	9
35	70.55<br>(52.21, 88.89)	99.91<br>(99.79, 100)	0.79<br>(0, 1.88)	8
40	70.01<br>(43.07, 96.95)	99.86<br>(99.62, 100)	1.21<br>(0, 3.44)	6
45	63.51<br>(15.36, 100)	99.91<br>(99.74, 100)	0.83<br>(0, 2.33)	4
50	56.01<br>(0, 100)	99.95<br>(99.71, 100)	0.49<br>(0, 2.59)	3
55	40.22<br>(0, 100)	100<br>(100, 100)	0<br>(0, 0)	2
60	60.00<br>(60.00, 60.00)	100<br>(100, 100)	0<br>(0, 0)	1
65	60.00<br>(60.00, 60.00)	100<br>(100, 100)	0<br>(0, 0)	1

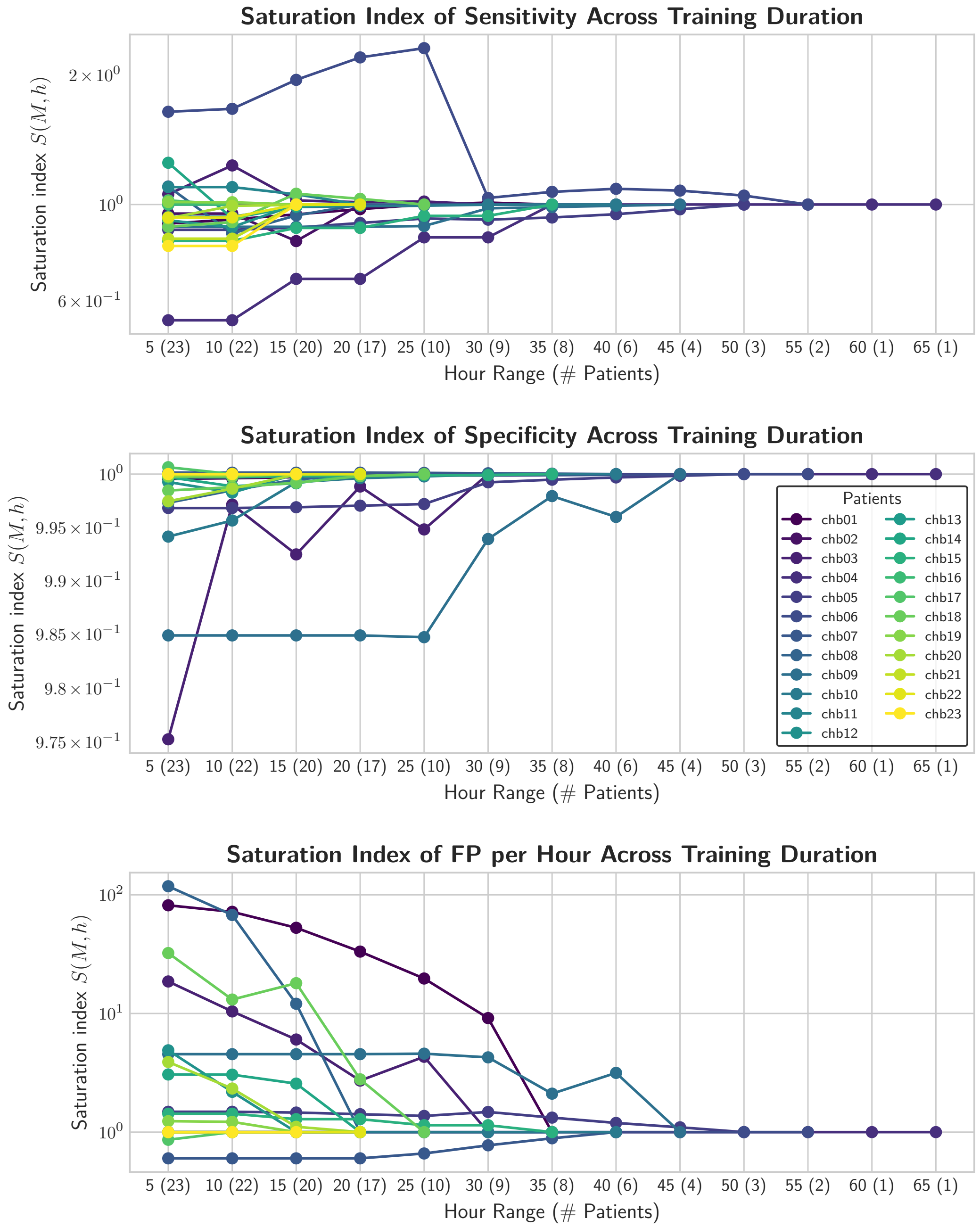

Figure S2. Individual patient variability in classifier metrics relative to asymptotic baselines. This figure illustrates the "saturation index" in classifier performance metrics for each patient, compared to their asymptotic baseline, which is determined by each individual's last available data point. The x-axis represents training duration in hours, showcasing how each patient's metrics evolve relative to their ending values. As the training duration increases, the number of represented patients decreases, indicating the varying extents of available data among individuals. Dataset: CHB-MIT.
